# Trauma-focused dialectical behavior therapy: study protocol for a randomized controlled multi-center trial in online and face-to-face formats

**DOI:** 10.1186/s40479-025-00294-3

**Published:** 2025-05-09

**Authors:** Ruben Vonderlin, Tali Boritz, Carola Claus, Büsra Senyüz, Saskia Mahalingam, Julia Schmelz, Silja Knolle-Ventjeer, Philip S. Santangelo, Ulrich W. Ebner-Priemer, Christian Schmahl, Jürgen Margraf, Tobias Teismann, Stefanie Lis, Nikolaus Kleindienst, Shelley McMain, Martin Bohus

**Affiliations:** 1 Department of Psychosomatic Medicine and Psychotherapy, Central Institute of Mental Health, Medical Faculty Mannheim, Heidelberg University, Mannheim, J5, 68159 Germany; 2 German Center for Mental Health (DZPG), Partner Site Mannheim - Heidelberg - Ulm, Mannheim, Germany; 3 Department of Psychology, York University, Toronto, ON Canada; 4 Centre for Addiction and Mental Health, Borderline Personality Disorder Clinic, Toronto, ON Canada; 5 Mental Health Research and Treatment Center, Ruhr-University Bochum, Bochum, Germany; 6 Center for Integrative Psychiatry – ZIP, Department of Psychiatry and Psychotherapy, Kiel, Germany; 7 Department of Behavioral and Cognitive Sciences, University of Luxembourg, Esch-sur-Alzette, Luxembourg; 8 Mental mHealth Lab, Faculty for Humanities and Social Sciences, Karlsruhe Institute of Technology (KIT), Karlsruhe, Germany; 9 Department of Psychiatry and Psychotherapy, Central Institute of Mental Health, Medical Faculty Mannheim, Heidelberg University, Mannheim, Germany; 10 Department of Clinical Psychology, Central Institute of Mental Health, Medical Faculty Mannheim, Heidelberg University, Mannheim, Germany; 11 Department of Psychiatry, University of Toronto, Toronto, Canada; 12 McLean Hospital, Harvard Medical School, Boston, MA USA

**Keywords:** Borderline personality disorder, Dialectical behavior therapy, Randomized controlled trial, TF-DBT

## Abstract

**Background:**

Borderline Personality Disorder (BPD) is a severe mental health condition that requires intensive psychotherapeutic treatment. Dialectical Behavior Therapy (DBT) is a specialized treatment approach for BPD with broad empirical evidence. However, as with other disorder-specific treatments, the effect sizes of the standard DBT approach are only modest and access to treatment is limited. To enhance the efficacy of DBT, we developed an adaptation called Trauma-Focused DBT (TF-DBT), which is based on the principles, treatment modes, and functions of DBT. The goal was to (i) condense and accelerate the core therapeutic processes of DBT and (ii) expand therapeutic strategies for addressing BPD symptoms beyond Stage I of DBT (i.e., focusing on behavioral dyscontrol). TF-DBT adopts an accelerated experiential approach that is phase-based in its delivery. It emphasizes the processing of maladaptive emotions related to a wide range of developmental relational trauma (i.e., experiences of traumatic invalidation, emotional abuse, bullying, sexual abuse, or neglect in childhood or adolescence).

**Aim:**

The primary aim of this study is to investigate the efficacy of this novel DBT adaptation (TF-DBT) compared to standard DBT (S-DBT) as developed by M. Linehan. We hypothesize that TF-DBT is superior to S-DBT on all BPD symptom measures. A second aim of the study is to investigate the efficacy of the delivery format of both treatments (i.e., online vs. face-to-face), with the hypothesis that online therapy is non-inferior to face-to-face treatment.

**Methods:**

This study will enroll *N* = 260 individuals diagnosed with BPD according to DSM-5. Participants will be randomly assigned to 12 months of outpatient TF-DBT or S-DBT in an online or face-to-face format.

**Discussion:**

The expected results might help to improve psychotherapy efficacy for BPD. Additionally, they will improve our understanding of the efficacy of online-delivered DBT treatments which might contribute to facilitating access to treatment.

**Trial registration:**

German Clinical Trials Register: registration number DRKS00031808, date of registration 04 July 2023. WHO Universal Trial Number: U1111-1273-3381.

**Supplementary Information:**

The online version contains supplementary material available at 10.1186/s40479-025-00294-3.

## Background

Borderline personality disorder (BPD) is a severe mental disorder characterized by significant instability in affect regulation, self-concept, interpersonal relationships, and behavioral control [1]. It affects approximately 2% of the adult population and is associated with chronic suicidality and recurrent self-harm, leading to significant individual suffering and extensive use of public health resources, particularly emergency and inpatient services [2, 3].

Clinical practice guidelines recommend psychotherapy as the first-line treatment for BPD [4, 5]. Dialectical Behavior Therapy (DBT) has amassed substantial empirical support among evidence-based interventions. Multiple meta-analytic studies have demonstrated its efficacy in improving various BPD-related symptoms, including emotional dysregulation, self-harm, and interpersonal dysfunction [6–9]. The latest Cochrane review, which analyzed 15 randomized controlled trials of DBT, found small to moderate effect sizes for overall BPD severity (*d* = −0.60), self-harm (*d* = −0.28), and psychosocial functioning (*d* = 0.36) compared to treatment as usual [10]. A recent DBT benchmarking study found pre-post effect-sizes of *d* = 0.82 as a DBT effectiveness benchmark, including RCTs and effectiveness studies [11]. While these findings underscore DBT’s effectiveness, they also highlight room for improvement.

Standard-DBT (S-DBT), originally developed by M. Linehan [12, 13], is a comprehensive, multi-component treatment approach. It integrates individual therapy, skills training groups, phone coaching, and therapist consultation teams to enhance patients’ behavioral skills, reduce maladaptive behaviors, increase emotional regulation, improve motivation, and support clinicians in delivering effective therapy. DBT follows a structured treatment hierarchy and operates within a dialectical framework that balances acceptance and change. Linehan’s model divides treatment into four stages, beginning with reducing life-threatening behaviors (Stage I) progressing to the processing of emotional difficulties (Stage II), skill-building in self-esteem and relationships (Stage III), and achieving deeper meaning and fulfillment (Stage IV).

To date, the majority of DBT research and clinical practice has focused on Stage I interventions. Consequently, guidance on addressing emotional dysregulation in Stage II and treatment strategies for the subsequent stages remains limited. Many therapists and clients struggle to transition beyond a focus on behavioural control, leaving a critical gap in treatment. The present study aims to enhance DBT by expanding therapeutic interventions to address treatment targets that span all stages of treatment, thereby accelerating treatment progression.

### Trauma-focused DBT: rationale and key modifications

To achieve this, we developed Trauma-Focused DBT (TF-DBT), which adopts an accelerated experiential approach by taking into account the key role that developmental relational trauma plays in the etiology and maintenance of BPD symptoms. A range of studies confirm that BPD develops from a wide range of adverse childhood experiences, including emotional abuse, neglect, bullying, and severe invalidation—forms of maltreatment collectively referred to as “developmental relational trauma” in this work [14–19]. Consistent with empirical research and Linehan’s original biosocial model of BPD, TF-DBT conceptualizes the core symptoms of BPD, i.e. emotion dysregulation, maladaptive self-concepts (e.g., self-hate, self-disgust), and interpersonal difficulties (e.g., heightened social threat or rejection sensitivity), as rooted in developmental relational trauma experiences (e.g., [20–25]). Linehan’s biosocial model of BPD [12, 13, 26] conceptualizes the disorder as arising from an interaction between biological predispositions, neurodevelopmental factors, and a range of early adverse experiences. Central to this model is the concept of an “invalidating environment,” where expressions of private experiences are met with inconsistent, dismissive, or extreme responses. Linehan later introduced the term “traumatic invalidation” to emphasize how repeated invalidation can threaten psychological integrity and contribute to BPD’s core symptoms, including intrusive trauma-related thoughts, heightened sensitivity to rejection, and difficulties in trusting others (Linehan [27]; p. 304).

Despite the central role of developmental relational trauma in the etiology and maintenance of BPD, trauma-related emotion processing is typically integrated after behavioural control is achieved in Stage II of DBT and has mainly focused on clients with comorbid PTSD. TF-DBT begins this work from the outset of treatment and integrates exposure-based techniques to guide trauma-related emotion processing within its central framework, targeting the range of adverse childhood experiences from traumatic invalidation to more severe forms of abuse, such as sexual abuse. The development of TF-DBT was inspired by DBT-PTSD, a phase-integrated model designed for individuals with PTSD related to childhood sexual abuse with and without co-occurring BPD [28]. DBT-PTSD has shown large pre-post effect sizes (*d* = 1.35) and significant reductions not only in PTSD, but also in BPD symptoms [29–31]. Encouraged by these findings, the treatment was expanded to include additionally BPD patients without co-occurring PTSD, retaining the trauma-focused approach to improve outcomes for BPD patients with diverse histories of developmental relational trauma, including experiences of invalidation, emotional abuse, or bullying. By integrating structured exposure therapy and emphasizing trauma processing within DBT’s existing framework, TF-DBT aims to more effectively target the core symptoms underlying BPD (i.e., emotion dysregulation, maladaptive self-concepts, interpersonal difficulties).

TF-DBT retains the core principles, treatment modalities, and functions of standard DBT while introducing the following key modifications:

1. Condensed treatment duration: We incorporate treatment targets relevant to all four stages of Linehan’s treatment and deliver this treatment within a one-year timeframe. There are four distinct treatment phases, each guided by specific goals, learning content, and therapeutic tasks.
2. Expanded focus on Wise Mind: We place a central focus on Linehan’s concept of Wise Mind and use an experiential approach to teaching this skill based on the cultivation of loving-kindness, compassion, empathetic joy, and serenity.
3. Broader application of structured exposure: We extend a structured exposure procedure to a range of developmental relational trauma experiences from traumatic invalidation, experiences of abandonment, neglect, and humiliation (e.g., bullying) to physical and sexual abuse.
4. Expansion of the emotion regulation module: While emotion regulation skills are taught in standard DBT, in TF-DBT these skills are expanded both in content and the experiential approach used to teach them. In TF-DBT, the concept of multiple emotions (i.e., the potential for experiencing conflicting and competing emotions) is added to the emotion regulation module. Further, an experience-based format supports clients in observing, describing and coping with multiple emotions in difficult day-to-day situations using the skills they have learned in the group.
5. Inclusion of social interaction training: In TF-DBT, interpersonal effectiveness is extended to include social interaction situation analyses and training. This experiential training involves skills-based role-playing of difficult day-to-day interpersonal situations.

### Improving access to care: evaluating online vs. face-to-face treatment delivery

There are barriers that limit access to DBT. As a specialized treatment, DBT is often available only at specialized centers in urban areas, making it difficult for many clients to access it [32, 33]. This issue is even more pronounced in developing and low- to middle-income countries [34]. Advancements in modern information technology have transformed the way psychotherapy can be delivered, leading to increased interest in the feasibility and effectiveness of internet-based and telehealth psychotherapeutic treatments as a means to improve access [35]. The COVID-19 pandemic further accelerated this shift, with DBT practitioners worldwide transitioning to telehealth delivery. Many clinicians in both healthcare centers and private practice now offer DBT remotely [36–38].

Despite this progress, concerns remain regarding the efficacy, safety, and feasibility of telehealth-delivered psychotherapy, particularly for patients with high levels of emotional and behavioral dysregulation [37, 39, 40]. There is limited empirical evidence comparing online psychotherapy to traditional face-to-face treatment, particularly in the context of DBT [41]. However, studies on other psychotherapeutic approaches—primarily cognitive behavioral therapy (CBT) for anxiety and affective disorders—have found no significant differences between online and face-to-face treatment formats [42, 43]. Preliminary findings from a feasibility trial to assess the feasibility, safety, and potential efficacy of online-delivered DBT in a controlled research setting are promising [44]. A total of 39 clients participated in one year of outpatient S-DBT treatment delivered online. Results indicated substantial pre-post effect sizes for BPD symptoms (*d* = 1.13 in the intent-to-treat sample (ITT) to 1.44 in the completer sample (ATP)) and quality of life (*d* = 0.65 in the ITT to 1.24 in the ATP). While the telehealth format was feasible and well-accepted, a relatively high dropout rate of 39% was observed. However, these results have not been compared to a face-to-face DBT group. Therefore, the present study includes a randomized comparison of online and face-to-face DBT across both treatments.

### Aims of the study

The primary aim of the present study is to compare the efficacy of Trauma-Focused DBT (TF-DBT) to a Standard-DBT program (S-DBT). Treatments are both one-year in length and are delivered in an outpatient setting. Both treatment models include individual therapy (1 h weekly) and skills group (2 h weekly), consultation team (1 h weekly), and phone coaching. A secondary aim is to compare the efficacy of online versus face-to-face treatments. Given the limited empirical evidence comparing online psychotherapy to traditional face-to-face treatment, particularly in the context of DBT [41, 44], the present study randomizes clients to in-person versus online treatment across both conditions.

## Methods

### Trial design & hypotheses

**Fig. 1:**
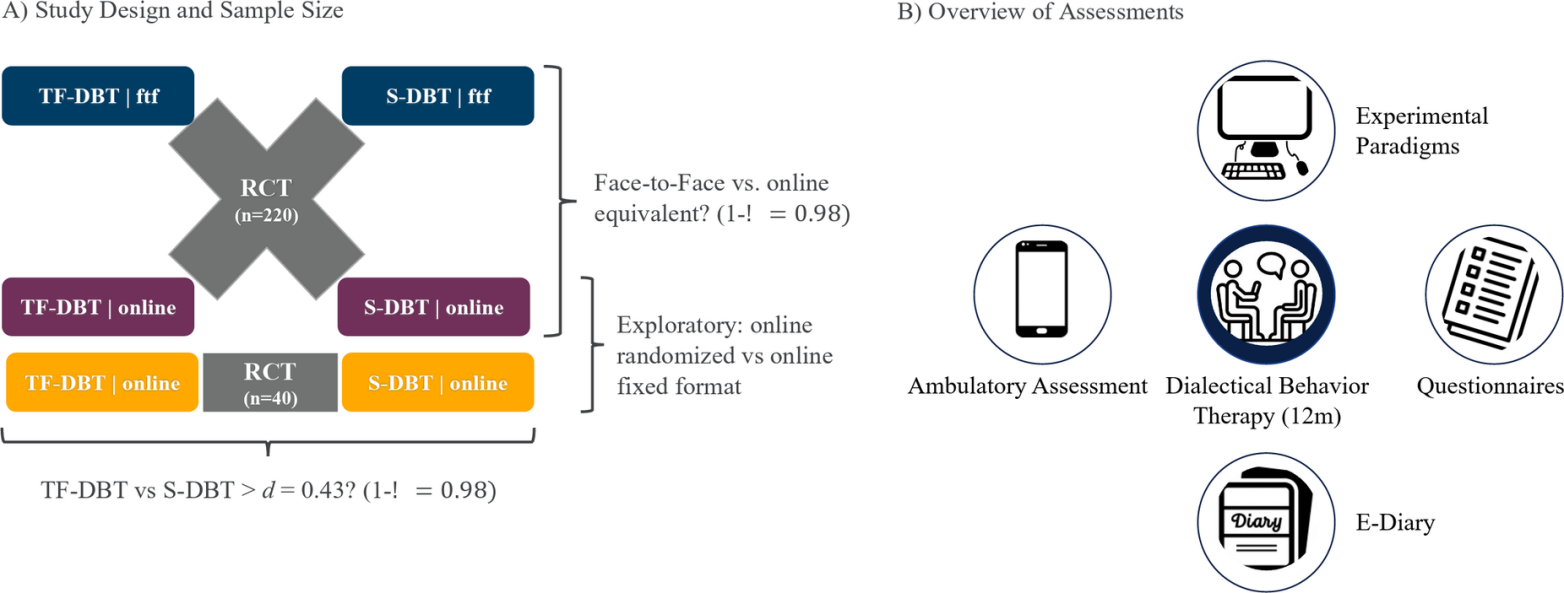
Study design and data assessments

The study will be conducted by an international research consortium at four different sites: the Central Institute of Mental Health in Mannheim, Germany; the Mental Health Research and Treatment Center at Ruhr University in Bochum, Germany; the Center for Integrative Psychiatry, University of Kiel, Germany; and the Center for Addiction and Mental Health in Toronto, Canada. The study will be conducted between 07/2023 (first patient in) and 03/2026 (last patient out). We aim to include *N* = 260 clients in the trial (*n* = 40 in Mannheim, Germany; *n* = 40 in Bochum, Germany; *n* = 40 in Kiel, Germany; *n* = 20 in Stuttgart, Germany; *n* = 80 in Toronto, Canada; and *n* = 40 German participants with > 1 h travel distance to a research site). Of those, 220 participants will be randomized to receive either TF-DBT or S-DBT in either online or face-to-face formats, resulting in 4 groups (see blue and purple groups in Fig. 1). In addition, 40 participants will be recruited with > 1 h travel distance to one of the research sites. This group will be established to exploratorily investigate potential benefits of the online format for patients who don’t have access to a face-to-face DBT treatment (see the orange group in Fig. 1). Participants in this group will be randomized to receive either TF-DBT or S-DBT in an online-format.

We hypothesize that the TF-DBT groups shows superior improvement in core BPD symptoms (i.e., emotion regulation, self-concept, and interpersonal interaction). We also hypothesize that an online treatment format is non-inferior to a face-to-face format. An overview of the study design is depicted in Fig. 1.

### Participants

We will include patients who (a) are between 18 and 65 years of age, (b) meet DSM-5 criteria for BPD, (c) are able to participate in online and face-to-face psychotherapy, (d) are able to participate in skills groups on a weekly basis, (e) are proficient in English (Toronto) or German (other sites), and d) consent to study participation. Patients will be excluded if they (a) meet diagnostic criteria for current psychotic disorder, current bipolar I disorder, have a body mass index of less than 17.5, severe substance use disorder or alcohol use disorder not in remission in the last 3 months, or dementia, (b) have an IQ less than 85, or (c) have received more than 8 weeks of standard DBT treatment within the last year. We will not exclude clients with a high suicidal risk.

### Recruitment procedure

Clients will be recruited in a staged procedure. First, a telephone screening is conducted to screen participants on eligibility criteria. Participants eligible for the study will be informed about potential participation in the study, and informed consent will be obtained. Next, the BPD section of the International Personality Disorder Examination Interview (IPDE) will be administered to assess BPD diagnosis [45], as well as the Mini International Neuropsychiatric Interview for DSM-5 (MINI, [46]) to assess comorbidities. Cognitive functioning will be assessed with the “Mehrfachwahl-Wortschatz-Intelligenztest” (MWT; [47]) in the German sample and the Test of Premorbid Functioning (TOPF; [48]) in the Canadian sample.

### Randomization and blinding

Randomization will be stratified by study center but is carried out centrally to avoid selection effects during randomization (allocation concealment in the sense of the CONSORT statement [49]). All patients fulfilling the inclusion/exclusion criteria and signing the informed consent form will be randomly assigned to one of the two treatment conditions (TF-DBT vs. S-DBT) and one of the two treatment formats (online vs. face-to-face) by random permutation of the pseudonymous study numbers in groups of 10. In addition, a minimization algorithm will be used during randomization to avoid imbalances with respect to co-occurring PTSD and ADHD as a main confounding factors for treatment success. The allocation sequence will be generated by a researcher who is not involved in the data collection (NK). The scientific staff conducting diagnostics and interviews will be blinded to the treatment allocation. However, a new blinded interviewer will be assigned if this rater becomes unblinded. Participants and therapists will not be blind, as blinding is usually not feasible during psychological treatments.

### Interventions and therapists

Both treatments adhere to the principles and guidelines of DBT established by M. Linehan. They also follow the same duration and intensity, incorporating individual sessions and closed skills groups over the course of one year of outpatient treatment. Telephone coaching is offered in both treatment arms and therapists in both treatment arms meet in a weekly consultation team.

#### TF-DBT (experimental group)

TF-DBT follows the dynamic hierarchy of DBT; whenever life-threatening or therapy-interfering behaviors occur, these targets are prioritized in treatment. Beyond that hierarchy, TF-DBT adopts a phase-based approach to structure and accelerate treatment progression. A more detailed description of TF-DBT is outlined in Supplementary Material 1: Appendix 1.

*Phase 1* is an *orientation and preparation phase* that consists of 20 sessions: In this phase, clients work on their individualized biosocial model of BPD, integrating biographical information assessed with a lifeline, significant relationship experiences as well as developmental relational trauma experiences. Furthermore, we aim to build motivation by formulating individual treatment goals directly linked to a life-worth-living. A crisis- and emergency plan is developed to help clients learn to understand and regain control over their most relevant dysfunctional behavior. Clients are introduced to the concept of Wise Mind as representing the attitudes of loving-kindness, compassion, empathetic joy and equanimity. Clients develop their own Wise Mind personal practice and listen to daily Wise Mind practices that are recorded by their therapists. Phase 1 closes with a case presentation by the client and therapist to the consultation team, which describes the case formulation and treatment plan for Phase 2 and beyond.

*Phase 2* is a *structured exposure phase* that consists of 15 sessions. In-sensu exposure techniques are used to reprocess emotion networks and maladaptive self-concepts routed in developmental relational trauma experiences that have threatened the client’s (i) need for belonging (e.g., through experiences of neglect, abandonment), (ii) need for status (e.g., through experiences of humiliation, bullying) or (iii) physical and sexual integrity of clients (e.g., through experiences of physical or sexual abuse) [25]. If clients report clinically relevant symptoms of PTSD that are linked to these traumata, the in-sensu exposure will start with the processing of this PTSD-relevant symptoms. If no comorbid PTSD is present, in-sensu exposure will focus on the core BPD features that are related to abandonment or humiliation.

The primary focus during Phase 2 sessions is on in-sensu exposure. Each session features a new in-sensu exposure, which is audiotaped. Clients listen to the most intense sequences of the audiotaped exposure sessions (10–15 min) at home between therapy sessions, which guides the out-of-session exposure and increases the number of exposure sequences. If clients exhibit high levels of behavioral avoidance, in-vivo exposures can be planned and assigned as homework. Toward the end of the exposure phase, clients complete a Wise Mind Exposure, a technique developed specifically for TF-DBT that combines mindfulness with exposure therapy. In the Wise Mind exposure, clients revisit their most distressing memories while maintaining a mindful stance grounded in self-compassion and equanimity, inspired by the Brahmavihara virtues. This technique aims to help clients process traumatic experiences from a more compassionate and balanced perspective, fostering integration and acceptance. This process can also help to restructure negative self-concepts, such as self-loathing or existential shame, encouraging clients to treat themselves with kindness and empathy.

*Phase 3* focuses on *Radical Acceptance of the past and building a life worth living*, consisting of 8 sessions. It cultivates the process of radical acceptance of the past and developing a life worth living. This phase helps clients reformulate their life goals based on their values and social, relational, and occupational contexts. Using this information, an individualized problem assessment is conducted to determine which specific skills can further help the client build a life worth living and foster social integration.

*Phase 4* is a brief *farewell* or termination phase that consists of 2 sessions, reflecting on what has been achieved, dealing with grief and the pain of parting, and planning the next steps after therapy.

A TF-DBT *skills group* runs in parallel with the phases of treatment outlined above. The emotion regulation module has been expanded to include trauma-related emotions and the concept of multiple emotions (acknowledging network theories of emotions); the interpersonal effectiveness module has been expanded to include interpersonal situation analyses and role-plays. The TF-DBT skills group consists of 40 sessions, each with unique content. Skills relate to the classical DBT modules of mindfulness, distress tolerance, emotion regulation, and interpersonal effectiveness.

#### Standard DBT (control group)

Similar to TF-DBT groups, the S-DBT groups take place over a one-year period and have the same length and number of sessions. The content of the S-DBT skills group is based on Linehan’s 2015 DBT manual [27]. The individual therapy sessions are organized by the highest priority targets informed by the clients’ diary cards; in other words, the sessions have an event-based focus. To remain adherent to Linehan’s 2015 DBT treatment guidelines, therapists were permitted to integrate an evidence-based exposure protocol into S-DBT (e.g., Prolonged Exposure) if indicated (i.e., if the client had a PTSD diagnosis) and if the client had achieved stabilization in Stage I targets (i.e., no self-harm or suicide attempts) (Linehan [27]; p. 45).

### Therapists, training, and treatment adherence

Therapists in both treatment arms were recruited because of their expertise in delivering DBT for BPD. All therapists had a minimum of one year delivering DBT. Additionally, all therapists received a minimum of three days of basic training in S-DBT. Therapists in the experimental group received two additional days of advanced training to teach all relevant adaptions that have been made in TF-DBT. The training and experience of the therapists were balanced across treatment groups. To ensure training quality, all therapists in the TF-DBT arm were required to have treated at least two clients during the pilot study. To ensure treatment fidelity, TF-DBT and S-DBT were not provided by the same therapists; therapists also attended separate weekly consultation teams, which included review of therapy videos. To assess adherence to the treatments, four sessions per therapist will be randomly selected and coded for treatment adherence. Adherence for S-DBT will be assessed using the Dialectical Behavior Therapy Adherence Coding Scale (DBT-ACS [50]), while adherence to TF-DBT will be assessed with an adapted version of the DBT-ACS that reflects the core modifications specific to TF-DBT.

### Assessments

**Fig. 2:**
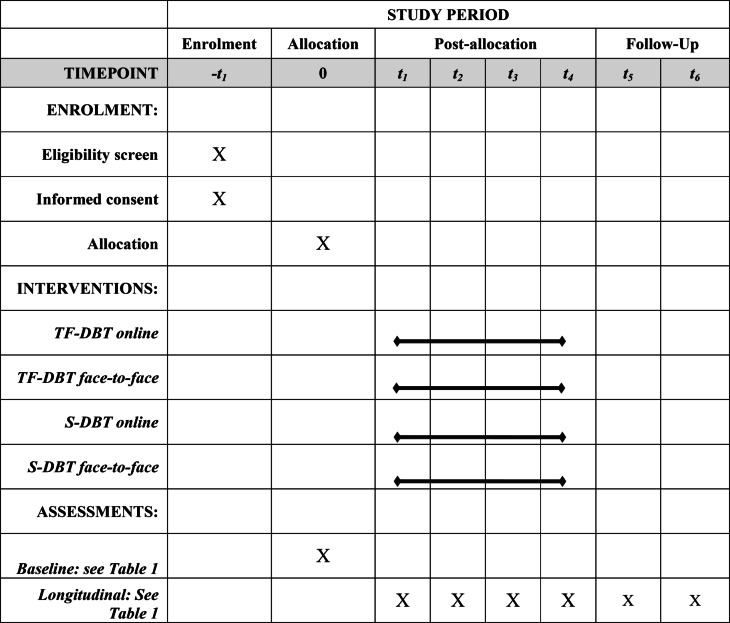
SPIRIT Schedule of enrolment and interventions for the protocol. Note. t1–3 months after treatment start, t2–6 months, t3–9 months, t4–12 months (post-assessment), t5–6 months follow-up, t6–12 months follow-up

Data will be collected through clinical interviews and self-assessment questionnaires before treatment and at 3, 6, 9, and 12 months after admission, as well as 6- and 12-months follow-up. The interviews are conducted by blind raters. Additional assessments contain experimental paradigms and ambulatory assessments at pre- and post-treatment (12 months). Furthermore, an electronic daily diary card - a basic tool in DBT – will be used to collect data. An overview of all assessment methods used is illustrated in Figs. 1 and 2. Table 1 depicts a list of all assessment instruments used.

**Table 1:** Assessments instruments and assessment times planned for the study. A detailed description of each instrument can be found in Supplementary Material 1: Appendix 2

Domain	Construct	Measure	Format	Acronym	Pre	0	3	6	9	12	18	24
Diagnostic Measures	BPD diagnosis	International Personality Disorder Exam (BPD subsection) [45]	Interview	IPDE	●						●	
	Comorbidities	Mini International Neuropsychiatric Interview DSM-5 [46]	Interview	MINI	●							
	Premorbid Functioning	Test of Premorbid Functioning [47, 48]	Interview	TOPF	●							
	ADHD	Adult ADHD Self-Report Scale [51]	Self-Report	ASRS	●							
	Personality Dimensions	Personality Inventory for DSM-5 [52, 53]	Self-Report	PID-5	●							
	Demographics	Demographic Data Survey	Self-Report	DDS	●							
Trauma Biography	Childhood Invalidation	Invalidating Childhood Environment Scale [54]	Self-Report	ICES	●							
	Childhood Trauma	Childhood Trauma Questionnaire [55]	Self-Report	CTQ	●							
	Complex Trauma	International Trauma Questionnaire [56]	Self-Report	ITQ	●							
	Embitterment	Posttraumatic Embitterment Disorder Questionnaire/Berner Embitterness Scale [57]	Self-Report	PTED/BEI	●							
	Unpredictability Childhood	Questionnaire of Unpredictability in Childhood [58]	Self-Report	QUIC	●							
	Bullying	Bullying Scale for Adults [59]	Self-Report	BSA	●							
BPD-related Psychopathology	BPD Symptoms	Borderline Symptom Liste - Interview	Interview	BSL-I		●	●	●	●	●	●	●
	BPD Symptoms	Borderline Symptom List 23 [60]	Self-Report	BSL-23	●	●	●	●	●	●	●	●
	Suicide Severity	Columbia Suicide Severity Scale [61]	Interview	C-SSRS		●	●	●	●	●	●	●
	Self-Harm	Deliberate Self-Harm Inventory [62, 63]	Self-Report	DSHI		●	●	●	●	●	●	●
	Emotion Regulation Difficulties	The Difficulties in Emotion Regulation Scale– 16 Item Version [64]	Self-Report	DERS-16		●	●	●	●	●	●	●
	Dissociation	Dissociative Symptoms Scale [65]	Self-Report	DSS		●	●	●	●	●	●	●
	Complex Trauma	International Trauma Questionnaire [56]	Self-Report	ITQ		●	●	●	●	●	●	●
General Psychopathology	General Psychiatric Symptom Distress	Symptoms Checklist 27 [66]	Self-Report	SCL-27		●	●	●	●	●	●	●
	Affective Suicidality	Acute Suicidal Affective Disturbance Interview [67]	Interview	ASAD-I		●	●	●	●	●	●	●
	Affective Suicidality	Acute Suicidal Affective Disturbance Questionnaire [67]	Self-Report	ASADI-L		●	●	●	●	●	●	●
	Severity Personality Disorder	Level of Personality Functioning Scale - Brief Form 2.0 [52]	Self-Report	LPFS-BF 2.0		●		●		●	●	●
Social Relationships and Affiliation	Loneliness	UCLA Loneliness Scale [68]	Self-Report	UCLA		●		●		●	●	●
	Social Connectedness	Social Connectedness Scale– Revised [69]	Self-Report	SCS-R		●		●		●	●	●
	Rejection Sensitivity	Rejection Sensitivity Questionnaire [70]	Self-Report	RSQ		●		●		●	●	●
	Need to Belong	Need to Belong Scale [71]	Self-Report	NBS		●		●		●	●	●
	Social Network Index	Social Network Index (+ Interpersonal Distance & Trust) [72]	Self-Report	SNI		●		●		●	●	●
Self-image and Self-esteem	Fear of Compassion	Fears of Compassion Scale [73, 74]	Self-Report	FCS		●		●		●	●	●
	Self-Esteem	Rosenberg Self-Esteem Scale [75]	Self-Report	RSES		●		●		●	●	●
	Self-Compassion	Self-Compassion Scale [76]	Self-Report	SCS		●		●		●	●	●
	Shame	Test of Self-Conscious Affect-3 [77]	Self-Report	TOSCA-3		●		●		●	●	●
	Aspects of Identity	Aspects of Identity Questionnaire [78, 79]	Self-Report	AIQ		●		●		●	●	●
Quality of Life and Wellbeing Outcomes	Functioning	Global Assessment of Functioning (Observation-Based) [80]	Interview	GAF		●	●	●	●	●	●	●
	Quality of Life	Recovering Quality of Life [81, 82]	Self-Report	ReQoL		●	●	●	●	●	●	●
	Psychosocial Functioning	Inventory of Psychosocial Functioning [83]	Self-Report	IPF		●		●		●	●	●
	Functioning– Social, Interpersonal, Global	WHO Disability Assessment Schedule 2.0–12-Item Version [84]	Self-Report	WHODAS 2.0–12		●		●		●	●	●
	Life Satisfaction	Quality of Life Enjoyment and Satisfaction Questionnaire– Short Form [85]	Self-Report	Q-LES-Q-SF		●		●		●	●	●
	Meaning of Life	Meaning of Life Questionnaire [86]	Self-Report	MLQ		●		●		●	●	●
	Mindfulness	Kentucky Inventory of Mindfulness Skills [87]	Self-Report	KIMS		●		●		●	●	●
Therapeutic Relationship and Acceptance Measures	Therapeutic Alliance	Working Alliance Inventory - Short Form, Client and Therapist [88]	Self-Report	WAI-S		● (S1-4)	●	●	●			
	Group Climate	Group Climate Questionnaire [89]	Self-Report	GCQ		● (S1-4)	●	●	●			
	Therapist Demographics	Therapist Demographic Questionnaire	Self-Report	TherDem		●						
	Acceptability of Intervention	Acceptability of Intervention Measure [90, 91]	Self-Report	AIM			●	●		●		
	Client Satisfaction	Client Satisfaction Questionnaire– 8 [92]	Self-Report	CSQ-8			●	●		●		
	Telehealth Usability	Acceptance and Use of Technology [93]	Self-Report	UTAUT			●	●		●		
Treatment Termination Measures	Reason for Treatment Termination	Research Early Termination for the Client*	Self-Report	RET-C								

Note: S1-S4 indicates that instruments are assessed in the first four session of individual therapy (WAI) and group therapy (GCQ)

*The RETC will be administered only if clients discontinue treatment prematurely

As compensation guidelines for participants differ in Canada and Germany, each site followed its own ethical standards of practice. At the German sites, participants did not receive financial compensation for data assessments while undergoing treatment. However, in cases where participants discontinue treatment prematurely but continue participating in research they receive €20 (US $21) for each subsequent assessment time point. At the Toronto site, all participants received CAD $15 (US $10.50) per assessment hour.

#### Primary outcome

*The Borderline Symptom List Interview (BSL-I*) was selected as the primary outcome of this trial, capturing the severity of BPD symptoms. The interview consists of 25 items assessing frequency (0 = never; 4 = very often) and associated distress (0 = no distress; 4 = most severe distress). Of these 25 items, four items additionally assess the consequences of behavioral symptoms (0 = no problematic behavior; 4 = behavior with extremely serious or life-threatening consequences). One additional item assesses psychosocial functioning in daily life (everyday life coping, social relationships, occupation,) and five items assess aspects of positive mental health. The BSL-I demonstrates good internal consistency within BPD samples (Cronbach’s α = 0.82), good interrater agreement (Kα = 0.76), as well as good criterion validity with high correlations with other established BPD self-report measures, like the BSL-23 (*r* = 0.83).

#### Secondary outcomes and potential mechanisms

As secondary outcomes, we will assess a broad range of outcomes and therapy processes, including (1) developmental relational trauma experiences (2), BPD-related psychopathology (3), general psychopathology (4), social relationships and affiliation (5), self-image and self-esteem (6), quality of life and wellbeing outcomes, as well as (7) therapeutic relationship and satisfaction with the intervention. The instruments used are depicted in Table 1, and a detailed description of each instrument can be found in Supplementary Material 1: Appendix 2.

In the present study, we primarily rely on the self-report and observer-based measures outlined above to assess change in BPD symptomatology and relevant domains. While validated psychometric instruments are considered the gold standard for assessing outcomes in intervention studies, they also provide a limited understanding of the psychological processes underlying change and may overlook treatment effects that manifest in clients’ daily lives. To expand the scope of this study, we aim to incorporate additional methods to capture clinical improvements and mechanisms of change beyond psychometric instruments. Specifically, we will employ experimental laboratory assessments of social information processing to directly evaluate potential psychological mechanisms of change and a prediction of treatment efficacy. Additionally, we will use ambulatory assessment methods to measure clinical outcomes and mechanisms of change in real-life settings. Experimental paradigms and Ambulatory Assessment procedures will be conducted at baseline, 2 to 4 weeks prior to the start of therapy, and at post-intervention, 2 to 4 weeks after the end of the intervention (12 months). Finally, to understand which psychopathological symptom clusters change in which phase of the treatment we will examine diary card data. This data is collected daily during treatment.

##### Experimental task battery 

Many experimental studies have identified alterations in social information processing (SIP) in BPD with the aim to understand impaired social functioning in these individuals. Alterations comprise self- and interpersonal functioning including cognitions, emotions and behaviours (e.g., [94–96]. However, studies are sparse that actually link these alterations in SIP to social functioning in BPD, to changes in the level of psychopathology after psychosocial interventions or to the stability of remission and recovery. Therefore, SIP and its changes after therapy will be assessed together with the participants’ appraisal of their social network as secondary outcomes using an experimental social-cognitive task battery and an egocentric social network survey. The central constructs of the task battery being assessed are social attitudes (value orientation), evaluation processes of social cues (attribution of emotions and trustworthiness to facial stimuli), inferring social causality (assessment of the likelihood of attributions in social scenarios), the experience of interpersonal closeness after simulated social interactions (partial cyberball paradigm with trust game) as well as the cognitive and emotional reactions to processes of self-evaluation. For a more detailed description, see Supplementary Material 1: Appendix 3.

##### Ambulatory assessment

The method of ambulatory assessments (AA) will be included to investigate emotional processing in clients’ daily lives. AA offers the possibility of repeated real-time assessments in patients’ natural habitat and, thus, the key advantages of examining dynamic processes with high ecological validity. AA data is used to examine disorder-relevant phenomena such as affect dynamics or stress reactivity [97, 98]. We hypothesize that individuals meeting the criteria for BPD, amongst others, suffer from intermittent implicit activations of cognitive-emotional networks, which deeply affect their day-to-day mental state. These maladaptive mental networks can be triggered by a variety of internal or external triggers or may result from dysfunctional social perceptions. We assume that the components of these maladaptive networks (cognitions, emotions, action tendencies, physiology) are highly individualized and closely linked. We further assume that activated maladaptive networks have an influence on implicit social evaluation processes. We aim to investigate whether TF-DBT, which specifically addresses these maladaptive networks, will reduce frequency and intensity of these networks in daily life circumstances. The AA protocol involves 4 days with 13 prompts per day assessing momentary cognitions, emotions, action tendencies and physiology and will be administered pre- and post-intervention. A more detailed outline of the procedure and materials can be found in Supplementary Material 1: Appendix 4.

##### Electronic diary card

The DBT diary card is an important therapeutic tool to promote patients’ self-reflection and to inform the therapist about the course of important symptoms (e.g., suicidal ideation, self-harm). In the past, this diary card has mainly been filled in paper-pencil to inform treatment processes and decisions. However, emerging research has demonstrated the utility of these data for research purposes (e.g., [99, 100]). Thus, in this study, we will administer the DBT Diary Cards electronically to use these daily data also for research purposes and to integrate them more easily into the online intervention. With this diary-card data, we aim to investigate the prediction of treatment response as well as premature therapy termination using machine learning algorithms [101, 102]. Furthermore, we aim to understand which symptom clusters change when during treatment, hypothesizing that dissociative symptoms, high suicidal urges, and behavioral dyscontrol change earlier in treatment, whereas improvements in maladaptive self-concepts, such as self-compassion, change later in treatment. The diary card will be administered on a daily level during the 12 months of treatment. A detailed description of the procedure and items used is outlined in Supplementary Material 1: Appendix 5.

### Statistical methods

#### Power analysis

The total sample size estimation of *N* = 260 is based on statistical power analyses regarding the intent-to-treat (ITT) sample. In comparing the treatment conditions (TF- DBT vs. S-DBT), a power of 1-*β* = 0.90 is aimed for detecting a minimum clinically relevant difference between the treatment conditions of *d* = 0.43, as recommended in the current Cochrane Review [10]. When comparing the treatment formats (online vs. face-to-face), an equivalence test with a power of 1-*β* = 0.90 is aimed for. The minimum relevant difference is also set at a standardized mean difference (Cohen’s *d*) of 0.43 in accordance with the current Cochrane review and the current Cochrane guidelines [10]. To achieve power, at least *n* = 230 participants are required. Because the psychotherapies investigated each require closed skills groups, participants are randomized into groups of 10. This results in a target number of cases of *N* = 260.

#### Statistical analyses

The analyses of the clinical efficacy of the treatments are carried out by modeling the primary outcome using mixed linear models (MLM). To compare the efficacy of the two groups, the interaction between treatment condition (TF-DBT vs. S-DBT) or treatment format (online vs. face-to-face) and time is tested for significance. To estimate the effect sizes, standardized mean differences (Cohen’s d) are calculated in TF-DBT before and after therapy as well as between the groups (TF-DBT vs. S-DBT) after therapy.

The main analyses include the evaluation of the ITT sample, which includes all patients who were randomized to a treatment in the study (randomized is analyzed). To analyze the ITT sample, including all participants who had been randomized to the treatment, we will impute missing data by multiple imputation procedures [103]. In addition, the main analyses are performed for all patients who complete the therapy (according to protocol).

#### Discontinuation and dropout policy

Completion according to protocol is defined as participation in at least 30 of the individual therapy sessions over a period of one year. This also applies if participants leave the study early, but at least after 30 sessions. A premature end of therapy is classified according to the following categories:

No-show (discontinuation before the first therapy session, therapy was not started: these patients are not included in the attrition rate); Early dropout (discontinuation within the first four therapy sessions); Withdrawal (if the therapist or treatment team decides that a participant should be withdrawn from treatment); Treatment Dropout (discontinuation of treatment after 5 th therapy session by patient); Research Dropout (participant withdraws consent to participate in the study). Patients who will be no longer available for study-related assessments will be classified as Lost to Follow-Up.

Patients who miss five consecutive weeks of individual therapy or group therapy are considered to have discontinued treatment. These patients are assigned to the dropout category.

Early Remission (Early successful symptom reduction, after 12 sessions at the earliest) is defined as a patient who no longer fulfills the diagnostic criteria for BPD on the IPDE (diagnostic remission assessed by blinded diagnosticians) and has a mean score on the BSL-23 of < 1.07 (this corresponds to no or mild residual symptoms according to Kleindienst et al. [104]) and has achieved all their treatment goals according to their own statement. To classify a participant as early remitted, the patient’s status must be agreed upon by the patient, therapist, and supervisor.

### Ethics, data management and dissemination

#### Research ethics approval

The study was approved by the ethical review committees from all sites: the University of Heidelberg (ID: 2023 − 518), Ruhr-University Bochum (ID: 865), Kiel University (ID: B262/23), and the Center for Addiction and Mental Health in Toronto (ID: 023–2023). A pre-registration was made in the German Register of Clinical Studies (DRKS00031808).

#### Consent to participate

All participants interested in the participation will receive verbal and written information from the study staff at the respective site. Prior to the start of the diagnostic assessments and before randomization, written consent must be obtained from all participants.

#### Data security and confidentiality

##### Interviews and questionnaires

All interviews and self-report measures will be collected and managed using REDCap electronic data capture tools hosted at CIMH for the German data and at CAMH for the Canadian data [105, 106]. REDCap is a secure, web-based software platform designed to support data capture for research studies. Participants will receive a study code prior to enrollment and data will be stored using this study code without containing any personal information. A separate key file with the assignment of study code and plain name is kept outside the REDCap landscape at the respective site and is only accessible by authorized project staff members, who are subject to the obligation of privacy. Documents with identifying information (e.g. consent forms) will be stored separately from the study data. The German data are centrally stored at the CIMH in Mannheim; the Canadian data are stored at the CAMH in Toronto.

##### Experimental tasks

The experimental tasks are programmed via Presentation (https://www.neurobs.com). The behavioral data with double coding are collected on study computers and stored on a server of the CIMH (the key is known only to the study staff).

##### Ambulatory assessment

The handling of the collected data is HIPPA-compliant: data are transferred in encrypted form from the electronic diaries to a server, where they are stored pseudonymously under the participants'study pseudonyms. The data stored on the server can be downloaded via a web frontend. Access to the web frontend is password protected. Data transfer from the server to the study director's browser is also encrypted.

#### Harms and crisis management

According to the European Medicines Agency Good Clinical Practice Guidelines, adverse events (AEs) and serious adverse events (SAEs) will be monitored during treatment in both treatment arms. All therapists will be routinely asked on a weekly basis whether a (S)AE has happened for each of their clients. If applicable, the research team will assess its intensity, outcome, and association to the treatment. SAEs will be reported to the respective ethics committee at each site. In addition, a Data Safety Monitoring Committee (DSMC) will be established, consisting of n = 3 independent researchers who are monitoring the safety of the trial. In order to protect participant safety, the DSMC is responsible to review the research protocol and plans for data safety and monitoring, conduct bi-annual evaluation of relevant safety data during the study and make recommendations concerning continuation, termination, or modifications of the study based on the observed beneficial or adverse effects. Accident and travel insurance was taken out for all participants in the study. There is also liability insurance for all therapists in the study.

#### Psychotropic medication protocol

There is currently no established pharmacological treatment protocol for BPD. However, discontinuation of medications would limit recruitment to a small number of patients with less severe pathology. Participants will be allowed to continue or receive psychotropic medication while participating in the trial since restricting medications would limit the generalizability and external validity of the study findings. Medication and medication changes will be documented weekly to calculate any differences in medication management between conditions. Therapists will submit weekly session reports and note any potential medication changes.

#### Protocol amendments

Any amendments to this study protocol must be documented in writing and receive approval from all authors. Additionally, subsequent changes must undergo review and approval by the relevant ethics committees. The most recent version of the protocol, including any amendments, will be updated and made accessible in the German Clinical Trials Register.

## Discussion

TF-DBT aims to improve the efficacy and efficiency of DBT for the treatment of BPD. This novel adaption aims to condense and accelerate the core therapeutic processes of DBT and expands therapeutic strategies to address and modify core BPD related to developmental relational trauma. The present study aims to examine the efficacy of TF-DBT compared to standard DBT and to compare the efficacy of TF-DBT and S-DBT delivered in online versus face-to-face formats. Findings from this study may have important clinical and training implications. By using a range of self-report, observational, experimental paradigms, and ambulatory assessment data from daily life, this study will provide deeper insights into the dynamics of BPD psychopathology and potential treatment effects, extending the understanding beyond traditional questionnaire and interview data.

## Trial status

The first cohort of patients at each site (*n* = 130) began treatment between July 2023 and December 2023. Recruitment for the second cohort (*n* = 130) is currently underway and is expected to be completed by March 2025.

## Supplementary Information

Supplementary Material 1: Appendix 1. Detailed description of Trauma-focused DBT (TF-DBT). Appendix 2. Detailed description of secondary outcomes and potential mechanisms. Appendix 3. Detailed description of the experimental task paradigm on social cognitive functioning. Appendix 4. Detailed description of the ambulatory assessment design. Appendix 5: Detailed description of electronic diary card use.

## Data Availability

No datasets were generated or analysed during the current study.

## Abbreviations

ADHD: Attention–deficit/hyperactivity disorder
AE: Adverse Event
BPD: Borderline Personality Disorder
CAMH: Center for Addiction and Mental Health
CBT: Cognitive Behavioral Therapy
CIMH: Central Institute of Mental Health
DBT: Dialectical Behavior Therapy
DBT-ACS: Dialectical Behavior Therapy Adherence Coding Scale
DBT-PTSD: Dialectical Behavior Therapy for Posttraumatic Stress Disorder
DSM: Diagnostic and Statistical Manual of Mental Disorders
DSMC: Data Safety Monitoring Committee
HIPPA: Health Insurance Portability and Accountability Act
HLM: Hierarchical Linear Modeling
ITT: Intention to treat
PTSD: Posttraumatic Stress Disorder
RedCap: Research Electronic Data Capture
SAE: Serious Adverse Event
S-DBT: Standard Dialectical Behavior Therapy
TF-DBT: Trauma Focused Dialectical Behavior Therapy
