# Supplementary material for: Trauma-focused dialectical behavior therapy: study protocol for a randomized controlled multi-center trial in online and face-to-face formats
Source: Borderline Personal Disord Emot Dysregul. 2025 May 9;12:15. doi: 10.1186/s40479-025-00294-3 (PMC12065370; doi:10.1186/s40479-025-00294-3)
Supplement: Supplementary file 1 — Supplementary Material 1: Appendix 1. Detailed description of Trauma-focused DBT (TF-DBT). Appendix 2. Detailed description of secondary outcomes and potential mechanisms. Appendix 3. Detailed description of the experimental task paradigm on social cognitive functioning. Appendix 4. Detailed description of the ambulatory assessment design. Appendix 5: Detailed description of electronic diary card use. [file 40479_2025_294_MOESM1_ESM.docx]

# Appendix

Appendix 1: Detailed description of Trauma-Focused DBT (TF-DBT)

Appendix 2: Detailed description of secondary outcomes and potential mechanisms

Appendix 3: Detailed description of the experimental task battery on social information processing and egocentric social networks

Appendix 4: Detailed description of the ambulatory assessment design

Appendix 5: Detailed description of electronic diary card use

# Appendix 1: Detailed description of Trauma-Focused DBT (TF-DBT)

TF-DBT was designed to address BPD individuals’ complex and heterogeneous problems with the assumption that they typically result from relational trauma, i.e. emotional abuse (e.g. invalidation, bullying), neglect, physical, or sexual traumatic experiences during childhood and adolescence. TF-DBT follows the dynamic hierarchy of DBT; whenever life-threatening or therapy-interfering behaviors occur, these targets are prioritized in treatment. Beyond that hierarchy, TF-DBT adopts a phase-based approach to structure and accelerate treatment progression. For this purpose, TF-DBT will be administered according to two sub-protocols: Clients without clinically relevant PTSD symptoms are treated according to the Traumatic invalidation (DBT-TI) protocol, in which the treatment focuses on enduring effects of relational trauma, like threat to status (e.g. bullying, humiliation) or threat to affiliation (e.g. rejection or abandonment; as described in the PAST framework (1)]), whereas clients with clinically relevant PTSD are treated according the established DBT-PTSD protocol focusing on physical or sexual traumatization (2). The decision according to which protocol a client will be treated is made after clinical consultation in a DBT consultation-team.

TF-DBT is organized into the same treatment modalities: individual therapy, skills groups, telephone coaching, and consultation teams. As an extension of Standard DBT (S-DBT), TF-DBT provides clients with a comprehensive self-help manual that includes readings and worksheets extending beyond skills training. Unlike S-DBT, which primarily focuses on the dysfunctional behavior patterns reported by the client, TF-DBT follows a predefined, sequential treatment protocol while maintaining the classic DBT hierarchy of prioritizing life-threatening and therapy-interfering behaviors whenever necessary (see Figure 1). The two sub-protocols (DBT-TI and DBT-PTSD) have many treatment components in common. However, they differ in the trauma-relevant aspects. Clients suffering from the consequences of sexual abuse in childhood or adolescence receive relevant information from the outset, and the focus of the skills is on dealing with guilt, shame and disgust. During the exposure phase in this subgroup, the focus is placed on dealing with the sexual trauma. There are also therapeutic contents that specifically consider dealing with trauma-related problems in sexuality, reducing the risk of re-traumatization and dealing with self-blame. For BPD clients who do not report a clinically relevant PTSD, the focus in the preparation phase is on identifying Core Emotional Networks (fear of abandonment and/or fear of humiliation; CEN), which are then processed intensively during the exposure phase. The skills groups are attended by both subgroups together.


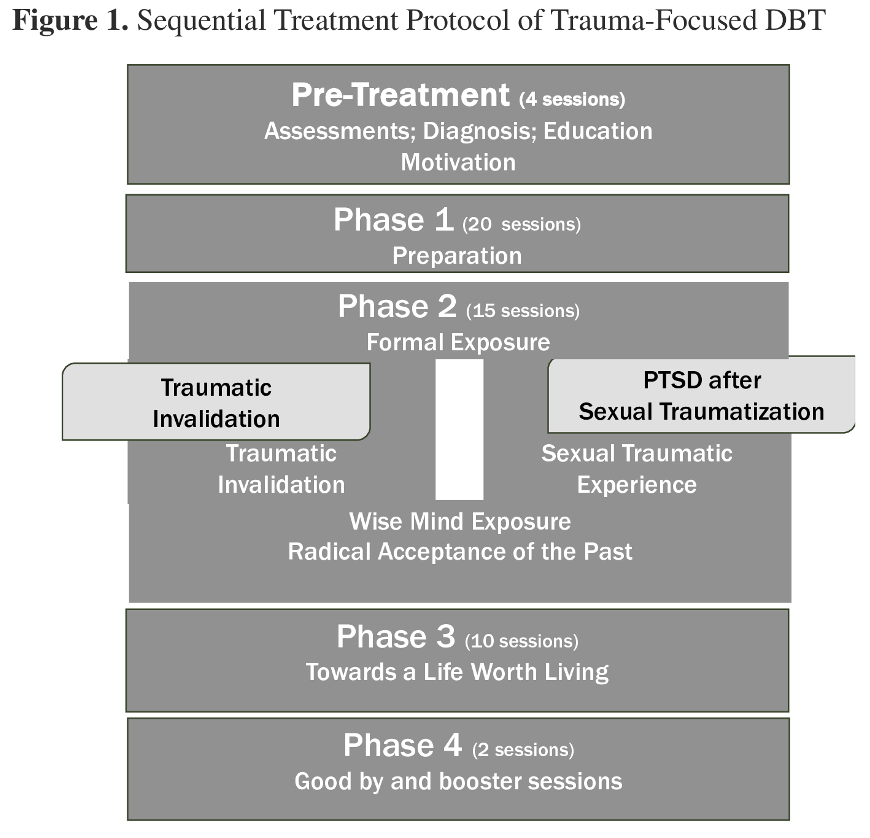


TF-DBT begins with a **preparation phase** (20 sessions; Phase 1). During phase 1, the focus is on psychoeducation and motivation, regaining control over dysfunctional behavior, development of an individualized patho-model (i.e. the identification of CEN or respectively the PTSD networks as driving forces of dysfunctional behaviors). Therapists first establish a crisis and emergency plan and introduce the TF-DBT diary card. With a lifeline, the therapist and client can obtain a brief overview of the client’s previous biographical experiences. A short introduction to the skills-concept also follows, as well as an introduction to “Wise-Mind”, an established DBT concept. The clients develop their own Wise-Mind personal practice and listen to daily imaginative self-instructions recorded by their therapists. These scripts define Wise-Mind as representing the attitudes of loving-kindness, compassion, empathetic joy and equanimity. As an optional tool, therapists explicitly anticipate and explore potential problems in the therapeutic relationship by analyzing significant relationship experiences of the client and discussing how these might affect the therapeutic relationship as well as the relationship with other members of the skills group (based on McCullough et al. (3)]). All therapists receive a checklist to assess the prominence of PTSD and determine whether it should be prioritized in treatment. This checklist is reviewed by the consultation team, which then decides on the appropriate treatment protocol (DBT-PTSD or DBT-TI). If a client’s PTSD diagnosis is prioritized, they follow the PTSD treatment model outlined by Bohus et al. (2). If PTSD is not the primary focus, clients instead work on their BPD model, addressing core emotional networks (CEN) such as fear of abandonment, and fear of social humiliation. These CEN include aversive emotions, cognitions, bodily sensations, and dysfunctional action urges that are defined as escape strategies. To establish a comprehensive case formulation, a model of “the old and new path” with the PTSD or BPD model as the old path, as well as values and goals to define the new path is developed. A key emphasis is on how PTSD and BPD symptomatology might prevent the client from living a life worth living. Based on this model the rational and concept for exposure is conveyed. At the end of this preparation phase, clients and their therapists present the treatment plan to the consultation team.

The **2nd treatment phase** consists of a formalized and structured 15-session exposure phase in which either traumatizing sexual events or experiences of social traumatization (traumatic invalidation) are reactivated and processed under controlled therapeutic conditions. Therapists are instructed to inhibit dissociative states or flashbacks during the exposure session, whereas clients learn that they can tolerate and process even highly aversive emotional states. The primary focus during sessions is on in-sensu exposure. However, therapists also encourage clients to complete in-vivo exposure as part of their homework, especially if they are avoiding certain behaviors or activities. Each session features a new in-sensu exposure, which is audiotaped. Clients listen to the most intense sequences of the audiotaped exposure sessions (10-15 minutes) at home between therapy sessions, which guides the out-of-session exposure and increases the number of exposure sequences. Progress of exposure is continuously monitored by a specifically developed software app (4). At the end of the exposure phase, we apply a newly developed procedure that helps to accept the traumatic past as a given and as an essential part of one's own biography. We call this technique Wise-Mind Exposure, which is a technique developed specifically for TF-DBT that combines mindfulness with exposure therapy. After completing the standard exposure work, clients revisit their most distressing memories while maintaining a mindful stance, grounded in self-compassion and equanimity, inspired by the Brahmavihara virtues. This technique aims to help clients process traumatic experiences from a more compassionate and balanced perspective, fostering integration and acceptance. In practice, it involves guided imagery, where clients visualize observing their younger selves with compassion, holding both the painful reality of their experiences and a broader understanding of the context. This process can also help to restructure negative self-concepts, such as self-loathing or existential shame, encouraging clients to treat themselves with kindness and empathy.

The **3rd treatment phase** (about 8 sessions) cultivates the process of radical acceptance of the past and further focuses on the development of a life worth living. TF-DBT offers pre-structured worksheets to reformulate one's life goals based on values and analyze the client’s social networks, satisfaction with relationships, and work situations. Using this information, an individualized problem assessment can be conducted to guide the next steps and the application of specific skills that support the individual client in building a life worth living and fostering social integration.

During the short **4th treatment phase** (two sessions), the focus is on reflecting on what has been achieved, dealing with grief and the pain of parting, and planning the next steps after therapy.

In addition to the adjustments in individual therapy, the content of **group** **skills training** was also modified from standard DBT. In the mindfulness module, there is a stronger emphasis on the concept of Wise-Mind. We extended this concept by integrating the four Buddhist Brahmavihara virtues: loving-kindness (maitrī/mettā), compassion (karuṇā), empathetic joy (muditā), and equanimity (upekṣā/upekkhā). Personalized imagery scripts are developed with the clients to change the negative self-concept in the long term. In the distress tolerance module, skills for dealing with dissociation have been added. The emotion regulation module has been expanded to include trauma-related emotions and the concept of multiple emotions (acknowledging network theories of emotions). Finally, the interpersonal effectiveness module has been expanded to include numerous exercises relevant to everyday life: At the core of this module is the interpersonal situation analysis, designed to promote experience-based processing and practice of interpersonal skills.

# Appendix 2: Detailed description of Secondary Outcomes and potential mechanisms

## Interviews

*Columbia Suicide Severity Scale (C-SSRS*; (5) is a clinician-administered interview used to evaluate suicidal ideation and behavior.

*Acute Suicidal Affective Disturbance* (*ASAD;* (6) is a clinician-administered interview used to evaluate a newly proposed diagnostic entity Acute Suicidal Affective Disturbance that characterizes rapid onset suicidal crises and is defined by four symptom groups (suicide intent, alienation, hopelessness and hyperarousal).

*Global Assessment of Functioning* (*GAF DSM-IV;* (7) is a rating scale used to assess an individual’s overall level of functional and psychological well-being, taking into account psychological symptoms and social functioning. As a numerical rating scale, scores can range from 0 to 100, divided into 10-point increments.

## Questionnaires

We will use a broad range of self-report questionnaires assessing different domains like (1) lifetime relational trauma experiences, (2) BPD-related psychopathology, (3) general psychopathology, (4) social relationships and affiliation, (5) self-image and self-esteem, (6) quality of life and wellbeing outcomes, as well as (7) therapeutic relationship and satisfaction with the intervention.

1. ***Lifetime relational trauma experiences***

The following questionnaires will be administered once at pre-treatment, after the diagnostic interviews to assess lifetime experiences of various types of traumatic experiences: Invalidating Childhood Environment Scale (ICES, (8)]), Childhood Trauma Questionnaire (CTQ, (9)]), International Trauma Questionnaire (ITQ, (10)]; German version (11)]), Questionnaire on Unpredictability in Childhood (QUIC, (12)], Bullying Scale for Adults (BSA, (13)]), Berner Embitterness Inventory (BEI, (14)]), Adult ADHD Self-Report Scale (ASRS, (15)]), Personality Inventory for DSM-5 (PID-5, (16, 17)]), demographic data, and medication.

1. ***BPD-related psychopathology***

*Borderline Symptom List – 23 (BSL-23*, (18)]; *German version* (19)]) is a self-report questionnaire that assesses BPD-specific symptoms. It contains 23 items rated on 5-point scale from 0 (not at all) to 4 (very strong). It will be administered at every assessment point. In addition, at pre- and post-intervention we will assess this questionnaire one and two weeks after the first assessment.

*Deliberate Self-Harm Inventory (DSHI*; (20, 21)]) is a self-report questionnaire designed to assess deliberate self-harm. It contains 17 items which collect information about frequency, severity, duration, and type of self-harming behavior.

*Difficulties in Emotion Regulation Scale – 16 item version (DERS-16*; (22)]). This measure will be used to probe client’s emotion regulation strategies. It consists of 16 items rated on a 5-point scale from 1 (almost never) to 5 (almost always).

*Dissociative Symptoms Scale (DSS;* (23)]) is a 28-item self-report questionnaire used to screen for dissociative symptoms. Items are rated on a scale from 0% (never) to 100% (always), with 10% increments in between.

*International Trauma Questionnaire* (*ITQ;* (10)]) is a 22-item self-report questionnaire used to assess the presence and severity of PTSD and CPTSD in individuals who have experienced traumatic events. Items cover re-experiencing, avoidance, negative alterations in cognition and mood, and hyperarousal.

1. ***General Psychopathology***

*Symptom Checklist-27 (SCL-27;* (24)]*)* is a self-report questionnaire that assesses general symptom distress. It contains a list of 27 clinical items rated on a scale from 0 (not at all) to 4 (extremely). It has 6 subscales: depressive symptoms, dysthymic symptoms, vegetative symptoms, agoraphobic symptoms, symptoms of social phobia, and symptoms of mistrust.

*Acute Suicidal Affective Disturbance* (*ASAD;* (6)]) is a self-report questionnaire assessing a newly proposed diagnostic entity that characterizes rapid onset suicidal intent and is defined by four symptom groups (suicide intent, alienation, hopelessness and overarousal).

*Level of Personality Functioning Scale - Brief Form 2.0 (LPFS-BF;* (25)]*)* measures personality functioning according to the Diagnostic and Statistical Manual of Mental Disorders, Fifth Edition's, alternative model of personality disorders, containing a total personality functioning score and two subscales (Self and Interpersonal).

1. ***Social relationships and affiliation***

*UCLA Loneliness Scale* (26) is a 20-item measure that assesses how often a person feels disconnected from others using a 4-point rating scale (1= never; 4 = always).

*Social Connectedness Scale Revised (SCS-R;* (27)]) is a 20-item self-report questionnaire used to measure social connectedness. Items are rated on a 6-point scale from 1 (strongly disagree) to 6 (strongly agree).

*Rejection Sensitivity Questionnaire (RSQ;* (28)]) is a self-report questionnaire used to assess rejection sensitivity, i.e., “the disposition to anxiously expect, readily perceive, and intensely react to rejection.” It contains 9 items rated on a 6-point scale from 1 (very unlikely or very unconcerned) to 6 (very likely or very concerned).

*Need to Belong Scale (NTB;* (29)]) is a 10-item self-report questionnaire used to assess need to belong (a desire to engage in interpersonal relationships). Items are rated on a 5-point scale from 1 (strongly disagree) to 5 (strongly agree).

*Social Network Index (SNI;* (30)]) is a 12-item self-report questionnaire which assesses participation in 12 types of social relationships, including relationships with a spouse, parents, parents-in-law, children, other close family members, close neighbours, friends, workmates, schoolmates, fellow volunteers, members of groups without religious affiliation, and religious groups.

1. ***Self-image and self-esteem***

*Rosenberg Self Esteem Scale* (RSES) is a 10-item self-report measure of global self-esteem. It consists of 10 statements related to overall feelings of self-worth or self-acceptance. The items are answered on a four-point scale ranging from strongly agree to strongly disagree.

*Self-Compassion Scale (SCS;* (31)]*)* is a self-report questionnaire used to measure self-compassion (including associated thoughts, emotions and behaviors). It contains 26 items rated on a 5-point scale from 1 (almost never) to 5 (almost always). The scale is comprised of 6 factors: self-kindness, self-judgment, common humanity, isolation, mindfulness and over-identification.

*Fears of Compassion Scale (FCS;* (32)]; *German Version* (33)]) contains 3 subscales measuring fear of compassion for self (compassion we have for ourselves when we make mistakes or things go wrong in our lives), fear of compassion from others (the compassion that we experience from others and flowing into the self), and fear of compassion for others (the compassion we feel for others, related to our sensitivity to other people’s thoughts and feelings).

*Test of Self-Conscious Affect-3 (TOSCA;* (34)]) is a self-report, scenario-based measure used to assess shame- and guilt-proneness. It contains 11 scenarios, each with 3 items rated on a 5-point Likert scale from 1 (not likely) to 5 (very likely). Scores are calculated for 3 subscales: shame self-talk, guilt self-talk, and blaming others.

*Aspects of Identity Questionnaire (AIQ-IV;* (35)]) is a 45-item self-report questionnaire used to assess identity orientations, i.e., “the relative importance that individuals place on various identity attributes or characteristics when constructing their self-definitions.” Items are rated on a 5-point scale from 1 (not important to my sense of who I am) to 5 (extremely important to my sense of who I am). The questionnaire is comprised of 4 subscales: Personal Identity Orientation, Relational Identity Orientation, Social Identity Orientation, and Collective Identity Orientation.

1. ***Quality of Life and Well being***

*WHO Disability Assessment Schedule 2.0 (WHODAS 2.0;* (36)]) *- 12-Item Version* is a comprehensive measure that assesses disability within the ICF biopsychosocial model of disability. It emphasizes the six domains of cognition, mobility, self-care, getting along with people, life activities and participation – including work-related disability.

*Inventory of Psychosocial Functioning* (IPF; (37)]) is a 64-item self-report questionnaire used to measure an individual’s functional impairment across various domains of life, including work, social and leisure activities, and personal relationships. The scale provides a total score as well as subscale scores for different areas of functioning.

*Recovering Quality of Life – 20 (ReQoL-20;* (38, 39)]) is a 20-item self-report questionnaire used to measure recovery-focused quality of life outcomes covering 7 themes: activity, hope, belonging and relationships, self-perception, well-being, autonomy, and physical health. Items are scored on a 5-point scale with the following response options: none of the time, only occasionally, sometimes, often, and most or all of the time.

*Quality of Life Enjoyment and Satisfaction Questionnaire-Short Form (Q-LES-Q-SF;* (40)]) is a 16-item self-report questionnaire used to measure life satisfaction and enjoyment by assessing satisfaction in the following domains: physical health, feelings, work, household duties, school/course work, leisure time activities, and social relations. Items are rated on a 5-point scale ranging from 1 (very poor) to 5 (very good).

*Meaning of Life Questionnaire (MLQ;* (41)]) is a 10-item self-report questionnaire used to assess the presence of meaning in life and the search for meaning in life. Items are rated on a 7-point scale ranging from 1 (absolutely untrue) to 7 (absolutely true).

*Kentucky Inventory of Mindfulness Skills (KIMS;* (42)]*)* is a self-report questionnaire used to assess mindfulness skills, including observing, describing, acting with awareness, and accepting (or allowing) without judgment. It contains 39 items rated on a 5-point scale ranging from 1 (never or very rarely true) to 5 (very often or always true).

1. ***Therapeutic alliance and treatment satisfaction***

*Working Alliance Inventory-Short Form (WAI-S;* (43)]) assesses the therapeutic alliance. This self-report measure has both a client version and a therapist version, each of which includes 12 items that the respondent rates on a 7-point scale from 1 (never) to 7 (always). Scores are calculated for one general alliance factor (range 12-84) as well as three core components of the alliance: agreement on goals, agreement on tasks, and personal bond. In addition to the regular time points, we will assess the therapeutic alliance after the first four individual therapy session to investigate the development of the therapeutic alliance at the beginning of therapy.

*Group Climate Questionnaire (GCQ;* (44)]) assesses the clients experience throughout group therapy. It consists of 12 items rated on a 7-point scale from 1 (not at all) to 7 (extremely), evaluating how the clients perceive engagement, potential conflicts and avoidance of the group as a whole. In addition, we adapted this questionnaire to ask clients to rate their perception of their own role in group, i.e. their own engagement, conflict with others or avoidance. In addition to the regular time points, we will assess the group cohesion after the first four group session to investigate the development of group cohesion at the beginning of therapy.

*Acceptance and Use of Technology (UTAUT;* (45)]) is a self-report questionnaire used to assess acceptance of digital health interventions based on the Unified Theory of Acceptance and Use of Technology and contains the dimensions of performance expectancy, effort expectancy, social influence, facilitating conditions and acceptance as well as internet experience, and internet anxiety. It consists of 20 items rated on a 5-point scale ranging from 1 (does not apply at all) to 5 (applies completely). The acceptance of the online psychotherapy will be assessed from therapist and client perspectives.

*Client Satisfaction Questionnaire-8 (CSQ-8;* (46)]) is a self-report questionnaire with items inquiring about respondents’ opinions and conclusions about services they have received or are currently receiving. Response options are based on a four-point scale.

*Acceptability of Intervention Measure (AIM;* (47, 48)]) is a 5-item scale that measures the perception among implementation stakeholders that a given treatment, service, practice, or innovation is agreeable, palatable, or satisfactory. It will be completed by therapists in both treatment arms.

# Appendix 3: Detailed description of the experimental task battery on social information processing and egocentric social networks

Many experimental studies have identified alterations in social information processing (SIP) in BPD with the aim to understand impaired social functioning in these individuals. Alterations comprise self- and interpersonal functioning including cognitions, emotions and behaviors (e.g., (49-51). However, studies are sparse that actually link these alterations in SIP to social functioning in BPD, to changes in the level of psychopathology after psychosocial interventions or to the stability of remission and recovery. Therefore, SIP and its changes after therapy will be assessed together with the participants’ appraisal of their social network as secondary outcomes using an experimental social-cognitive task battery and an egocentric social network survey. The central constructs being assessed are social attitudes (value orientation), evaluation processes of social cues (attribution of emotions and trustworthiness to facial stimuli), inferring social causality (assessment of the likelihood of attributions in social scenarios), the experience of interpersonal closeness after simulated social interactions (partial cyberball paradigm with trust game) as well as the cognitive and emotional reactions to processes of self-evaluation.

## Paradigms of the task battery

***Social value orientation***

To assess an individual’s social value orientation, we use the slider task of Murphy, Ackermann and Handgraaf (52). Similarly to a dictator game, participants divide fictitious money between themselves and an imaginary partner. To assess discrepancies in the experience of interpersonal values between oneself and others, the participants rate their expectation of the social value orientation of other people with an analogous task. In this task, participants are asked to assess their expectation of which allocations another, unfamiliar person would choose. Based on the assessments, the value orientation is calculated along a continuum from competitive to altruistic value orientation in both tasks. Previous studies have shown that individuals with BPD describe a prosocial orientation for themselves while assessing others as more individualistic (53). Since individuals high in BPD features have been shown to attribute a particularly high importance to fairness (54), the participants will evaluate how strongly they are concerned about justice and how they would expect the concern about justice others would experience by using the short form of the Justice Sensitivity Index (55).

***Social evaluation processes***

Emotion recognition and trustworthiness judgments as more complex social evaluation processes are assessed by evaluating the intensity of emotions (joy, anger) and trustworthiness in facial stimuli with varying emotional expression. Participants will see faces of different people who show a positive (joy) or negative (anger) facial expression and rate the intensity of the emotions and the trustworthiness of the faces, as well as their confidence their own evaluation. Studies by our research group showed that BPD patients attribute a lower intensity of joy and trustworthiness to positive faces and experience a higher uncertainty in their own assessment, which correlate with the extent of subjectively experienced social isolation, especially when evaluating positive emotions (56-58).

***Dysfunctional self-referential attributions***

To assess alteration in expectations of social causality, participants estimate the likelihood of positive, neutral and negative explanations in self-related positive and negative social scenarios. As an experimental control, the participants perform the same evaluations for other-related scenarios. In addition to the rated likelihood, which serves as the main dependent variable, participants are also asked to rate the valence of their emotional response for the different attributions in case they would be true A preliminary study has shown that, in contrast to healthy individuals, BPD patients rate the likelihood of explanations in self- and other-related scenarios differently, with a lower probability rating for positive attributions and a higher probability rating for negative attributions in self-related scenarios. This is consistent with studies that show that BPD patients tend to a maladaptive attributional style (e.g., (59)]) and report self-esteem-deprecating explanations for the behavior of others (60, 61).

***Appraisal of interaction behavior***

To assess the experience of social belonging during standardised social interactions, participants rate the experience of social belonging after a virtual ball throwing game with two players, one of whom includes the participant and the other excludes them. This ‘partial’ Cyberball paradigm is a recently developed modification of the long established Cyberball paradigm which allows to investigate behavioural responses to inclusion and exclusion during the game by changes in the passing preference (62). After the game, participants rate the strength of feeling included in the game by each of the two teammates. In addition to this dependent variable capturing the emotional response towards both teammates, we assess a cognitive component (rating of the percentage of ball throws received) and behavioral effects on trust towards the teammates (one-shot trust game played with each teammate).

***Effects of Self-referential processing on emotions and cognition***

For the experimental induction of self-referential processing such as self-awareness, self-reflection and self-assessment, we use the experimental paradigm by Biermann, Schulze (63). Here, participants answer standardised questions about facial images of their own person. After this confrontation with their own picture, participants are asked to assess their emotional response by rating the intensity of different emotions, which constitutes the main dependent variable of the paradigm. Additionally, participants complete a cognitive task (CPT-AX), which is used - in addition to the explicit effects of the experimental manipulation on the emotional state – as a measure of implicit effects of self-evaluation processes on cognitive performance. As an experimental control, participants are asked to rate the faces of strangers in two equivalently structured task blocks. Biermann, Schulze (63) found that BPD patients experience a higher degree of disgust after confrontation with their own faces.

***Egocentric social network analyses survey***

The participants’ social functioning will be assessed with an individualised approach by egocentric social network analysis. Egocentric networks allow for an individualised assessment of an individual’s social environment by taking both the relationships between the participant (ego) and the members of their network (alters) as well as the relationships between the alters into account. Previous studies have shown its suitability to uncover changes in social functioning in BPD (e.g., (64, 65)]). The patients’ characterization of their networks revealed more frequent break-ups, more negative and less positive ego-alter relationship and a lower influence of structural alter attributes on ego-alter relationships (66, 67). Pilot data from our group showed that, in particular, BPD patients' satisfaction with the relationships within their networks was related to the level of loneliness they experienced.

In the current study, participants (egos) are asked to set up the social network of the 10 most important people (alters) during the last three months of their lives. They will characterize the alters in terms of objective characteristics such as gender and social role. Relationships between ego and alters will be assessed in regard to importance, closeness, frequency of contact, support and conflict, and satisfaction in the various facets of the ego-alter relationship. As reciprocity is an important aspect in the experience of social relationships, participants will also assess the relationships from the perspective of the different members of the network**.** To measure the structure of the network, participants assess how close they think the members of the network are to each other. The social network analyses will focus on the composition and structure of the networks, as well as the interplay between structural attributes of the alters and the ego-alter relationships. Combining social network features and social information processing will provide insight into the mechanism underlying alterations in the client’s social network. Moreover, it will allow investigation into whether TF-DBT is superior to S-DBT in improving the clients’ appraisal of the social relationships within their social networks and whether changes in social information processing are associated to these changes.

# Appendix 4: Detailed description of the ambulatory assessment design

The method of ambulatory assessments (AA) will be included to investigate emotional processing in clients’ daily life. AA offers the possibility of repeated real-time assessments in patients’ natural habitat and, thus, the key advantages of examining dynamic processes with high ecological validity. AA data is used to examine disorder-relevant phenomena such as affect dynamics or stress reactivity (68, 69). We hypothesize that individuals meeting criteria for BPD, amongst others, suffer from intermittent implicit activations of cognitive-emotional networks, which deeply affect their day-to-day mental state. These maladaptive mental networks can be triggered by a variety of internal or external triggers, or may result from dysfunctional social perceptions. We assume that the components of these maladaptive networks (cognitions, emotions, action tendencies, physiology) are highly individualized and closely linked. We further assume that activated maladaptive networks have influence on implicit social evaluation processes. We aim to investigate whether TF-DBT, which specifically addresses these maladaptive networks, will reduce frequency and intensity of these networks in daily life circumstances. The AA protocol involves 4 days with 13 prompts per day assessing momentary cognitions, emotions, action tendencies and physiology and will be administered pre- and post-intervention.

## Procedure

We will assess within-subject dynamics of emotions, distress, self-esteem, cognitions, action tendencies, and trigger experiences in daily life. Participants will be asked to repeatedly answer questions presented on their smartphone device during their day-to-day activities across two time points over the course of the study period at pre- and post-treatment. At each of the two AA time points, data collection will occur over four days, during which the patients engage in their daily lives. During these four days, participants will be asked to answer a series of questions presented on their smartphone around 30 questions 13 times per day from 9 AM trough 22 PM in approximately one-hour intervals using the movisensXS software (movisens GmbH, Karlsruhe).

## Materials

Participants are asked about their current emotional state (e.g., happy; ashamed; lonesome etc.), cognitions (e.g., I am stupid; worthless etc.), bodily sensations (e.g., aversive tension, analgesia, emptiness etc.), action urges (e.g., self-harm, substance abuse etc.) as well as positive and negative events that might have worked as triggers. For all of these categories, we will provide a set of pre-written items, that all participants will receive, but patients may also add and rate their own items to these categories that represent their own individual network. In addition, we assess current self-esteem related to these networks based on the 4-item version of the Rosenberg Self-Esteem Scale (RSES; (70)]). To control for confounding variables, patients complete morning (at 9 AM) and evening (at 22 PM) questionnaires to assess sleep quality or unusual positive/negative incidents.

# Appendix 5: Detailed description of electronic diary card use

The DBT diary card is an important therapeutic tool to promote patients’ self-reflection and to inform the therapist about the course of important symptoms (e.g., suicidal ideation, self-harm). In the past, this diary card has mainly been filled in paper-pencil to inform treatment processes and decisions. However, emerging research has demonstrated the utility of these data for research purposes (e.g. (71, 72)). Thus, in this study, we will administer the DBT Diary Cards electronically to use these daily data also for research purposes and to integrate it more easily into the online intervention. With this diary-card data we aim to investigate the prediction of treatment response as well as premature therapy termination using machine learning algorithms (73, 74). Furthermore, we aim to understand which symptom clusters change when during treatment, hypothesizing that dissociative symptoms, high suicidal urges and behavioral dyscontrol change earlier in treatment, whereas improvements in maladaptive self-concepts, such as self-compassion, change later in treatment. The diary card will be administered on a daily level during the 12 months of treatment.

## Method

We will use the software STATUS from Vacay GmbH (https://vacay.dev) that has been developed to implement real-time feedback loops between therapists and clients during the therapy process (73, 74). Patients are able to install an app on their personal smartphone to answer the diary cards daily. Therapists can access a platform in their browser to view their patients' assessments throughout therapy.

In the TF-DBT diary card, the following items will be assessed: daily: pleasant/unpleasant events, misery, self-compassion, suicidal ideation, sleep quality, dissociation, trust in therapy, treatment goals, used skills, physical exercise, therapy homework, Wise-Mind exercises, as well as dysfunctional behaviors (urge and action). On a weekly basis, we track changes in medication, most (un-)pleasant events of the week, as well as utilization of crisis coaching.

In the S-DBT diary card the following items will be assessed: daily: urges to use substances, commit suicide and self-harm, dysfunctional behavior, emotions, substance abuse, skill use. On a weekly basis, we track the most important event of the week, a detailed skill-diary card, medication changes and utilization of crisis coaching.

# References

1. Neuner F. Physical and social trauma: Towards an integrative transdiagnostic perspective on psychological trauma that involves threats to status and belonging. Clinical Psychology Review. 2023;99:102219.

2. Bohus M, Schmahl C, Fydrich T, Steil R, Müller-Engelmann M, Herzog J, et al. A research programme to evaluate DBT-PTSD, a modular treatment approach for Complex PTSD after childhood abuse. Borderline Personality Disorder and Emotion Dysregulation. 2019;6:1-16.

3. McCullough Jr JP, Schramm E, Penberthy JK. CBASP as a distinctive treatment for persistent depressive disorder: Distinctive features. London: Routledge; 2014.

4. Görg N, Priebe K, Deuschel T, Schüller M, Schriner F, Kleindienst N, et al. Computer-assisted in Sensu exposure for posttraumatic stress disorder: development and evaluation. JMIR Mental Health. 2016;3(2):e5697.

5. Posner K, Brent D, Lucas C, Gould M, Stanley B, Brown G, Mann J. Columbia-suicide severity rating scale (C-SSRS). 2008 ed. New York, NY: Columbia University Medical Center; 2008.

6. Tucker RP, Michaels MS, Rogers ML, Wingate LR, Joiner Jr TE. Construct validity of a proposed new diagnostic entity: Acute Suicidal Affective Disturbance (ASAD). Journal of Affective Disorders. 2016;189:365-78.

7. Aas IM. Guidelines for rating global assessment of functioning (GAF). Annals of General Psychiatry. 2011;10:1-11.

8. Robertson CD, Kimbrel NA, Nelson-Gray RO. The Invalidating Childhood Environment Scale (ICES): psychometric properties and relationship to borderline personality symptomatology. Journal of Personality Disorders. 2013;27(3):402-10.

9. Bernstein DP, Fink L, Handelsman L, Foote J. Childhood Trauma Questionnaire (CTQ) [Database Record]. APA PsycTests. 1994.

10. Cloitre M, Shevlin M, Brewin CR, Bisson JI, Roberts NP, Maercker A, et al. The International Trauma Questionnaire: Development of a self‐report measure of ICD‐11 PTSD and complex PTSD. Acta Psychiatrica Scandinavica. 2018;138(6):536-46.

11. Christen D, Killikelly C, Maercker A, Augsburger M. Item response model validation of the German ICD-11 International Trauma Questionnaire for PTSD and CPTSD. Clinical Psychology in Europe. 2021;3(4).

12. Glynn LM, Stern HS, Howland MA, Risbrough VB, Baker DG, Nievergelt CM, et al. Measuring novel antecedents of mental illness: the Questionnaire of Unpredictability in Childhood. Neuropsychopharmacology. 2019;44(5):876-82.

13. Haidl TK, Schneider N, Dickmann K, Ruhrmann S, Kaiser N, Rosen M, et al. Validation of the Bullying Scale for Adults-Results of the PRONIA-study. Journal of psychiatric research. 2020;129:88-97.

14. Znoj H, Abegglen S, Buchkremer U, Linden M. The embittered mind. Journal of Individual Differences. 2016.

15. Kessler RC, Adler L, Ames M, Demler O, Faraone S, Hiripi E, et al. The World Health Organization Adult ADHD Self-Report Scale (ASRS): a short screening scale for use in the general population. Psychological Medicine. 2005;35(2):245-56.

16. Krueger RF, Derringer J, Markon KE, Watson D, Skodol AE. Initial construction of a maladaptive personality trait model and inventory for DSM-5. Psychological medicine. 2012;42(9):1879-90.

17. Bach B, Kerber A, Aluja A, Bastiaens T, Keeley JW, Claes L, et al. International assessment of DSM-5 and ICD-11 personality disorder traits: toward a common nosology in DSM-5.1. Psychopathology. 2020;53(3-4):179-88.

18. Bohus M, Kleindienst N, Limberger MF, Stieglitz R-D, Domsalla M, Chapman AL, et al. The short version of the Borderline Symptom List (BSL-23): development and initial data on psychometric properties. Psychopathology. 2009;42(1):32-9.

19. Wolf M, Limberger MF, Kleindienst N, Stieglitz R-D, Domsalla M, Philipsen A, et al. Kurzversion der borderline-symptom-liste (BSL-23): Entwicklung und überprüfung der psychometrischen eigenschaften. PPmP-Psychotherapie· Psychosomatik· Medizinische Psychologie. 2009;59(08):321-4.

20. Gratz KL. Measurement of deliberate self-harm: Preliminary data on the Deliberate Self-Harm Inventory. Journal of Psychopathology and Behavioral Assessment. 2001;23:253-63.

21. Fliege H, Kocalevent R-D, Walter OB, Beck S, Gratz KL, Gutierrez PM, Klapp BF. Three assessment tools for deliberate self-harm and suicide behavior: evaluation and psychopathological correlates. Journal of Psychosomatic Research. 2006;61(1):113-21.

22. Bjureberg J, Ljótsson B, Tull MT, Hedman E, Sahlin H, Lundh L-G, et al. Development and validation of a brief version of the difficulties in emotion regulation scale: the DERS-16. Journal of Psychopathology and Behavioral Assessment. 2016;38:284-96.

23. Carlson EB, Waelde LC, Palmieri PA, Macia KS, Smith SR, McDade-Montez E. Development and validation of the Dissociative Symptoms Scale. Assessment. 2018;25(1):84-98.

24. Kuhl HC, Hartwig I, Petitjean S, Müller-Spahn F, Margraf J, Bader K. Validation of the Symptom Checklist SCL-27 in psychiatric patients: Psychometric testing of a multidimensional short form. International Journal of Psychiatry in Clinical Practice. 2010;14(2):145-9.

25. Weekers LC, Hutsebaut J, Kamphuis JH. The Level of Personality Functioning Scale‐Brief Form 2.0: Update of a brief instrument for assessing level of personality functioning. Personality and Mental Health. 2019;13(1):3-14.

26. Russell DW. UCLA Loneliness Scale (Version 3): Reliability, validity, and factor structure. Journal of Personality Assessment. 1996;66(1):20-40.

27. Capanna C, Stratta P, Collazzoni A, D’Ubaldo V, Pacifico R, Di Emidio G, et al. Social connectedness as resource of resilience: Italian validation of the Social Connectedness Scale-Revised. Journal of Psychopathology. 2013;19:320-6.

28. Berenson K, Gyurak A, Downey G, Ayduk O, Mogg K, Bradley B, Pine D. Rejection sensitivity RS-adult questionnaire (a-RSQ). Measurement Instrument Database for the Social Science. 2013.

29. Leary MR, Kelly KM, Cottrell CA, Schreindorfer LS. Construct validity of the need to belong scale: Mapping the nomological network. Journal of Personality Assessment. 2013;95(6):610-24.

30. Cohen S, Doyle WJ, Skoner DP, Rabin BS, Gwaltney JM. Social ties and susceptibility to the common cold. Jama. 1997;277(24):1940-4.

31. Neff KD. The development and validation of a scale to measure self-compassion. Self and Identity. 2003;2(3):223-50.

32. Gilbert P, McEwan K, Matos M, Rivis A. Fears of compassion: Development of three self‐report measures. Psychology and Psychotherapy: Theory, research and practice. 2011;84(3):239-55.

33. Biermann M, Bohus M, Gilbert P, Vonderlin R, Cornelisse S, Osen B, et al. Psychometric properties of the German version of the fears of compassion scales. Clinical Psychology & Psychotherapy. 2021;28(1):137-49.

34. Tangney JP, Dearing RL, Wagner PE, Gramzow R. Test of Self-Conscious Affect–3 (TOSCA-3) [Database Record]. 2000.

35. Cheek JM, Briggs SR. Aspects of Identity Questionnaire (AIQ-IV). Measurement instrument database for the social science. 2013.

36. Üstün TB, Chatterji S, Kostanjsek N, Rehm J, Kennedy C, Epping-Jordan J, et al. Developing the World Health Organization disability assessment schedule 2.0. Bulletin of the World Health Organization. 2010;88:815-23.

37. Bovin MJ, Black SK, Rodriguez P, Lunney CA, Kleiman SE, Weathers FW, et al. Development and validation of a measure of PTSD-related psychosocial functional impairment: The Inventory of Psychosocial Functioning. Psychological Services. 2018;15(2):216.

38. Keetharuth AD, Brazier J, Connell J, Bjorner JB, Carlton J, Buck ET, et al. Recovering Quality of Life (ReQoL): a new generic self-reported outcome measure for use with people experiencing mental health difficulties. The British Journal of Psychiatry. 2018;212(1):42-9.

39. Grochtdreis T, König H-H, Dams J. Recovering Quality of Life: Deutsche Übersetzung eines Fragebogens zur Erfassung der gesundheitsbezogenen Lebensqualität von Menschen mit psychischen Erkrankungen. Das Gesundheitswesen. 2021;83(07):538-40.

40. Stevanovic D. Quality of Life Enjoyment and Satisfaction Questionnaire–short form for quality of life assessments in clinical practice: A psychometric study. Journal of Psychiatric and Mental Health Nursing. 2011;18(8):744-50.

41. Steger MF, Frazier P, Oishi S, Kaler M. The meaning in life questionnaire: assessing the presence of and search for meaning in life. Journal of Counseling Psychology. 2006;53(1):80-93.

42. Baer RA, Smith GT, Allen KB. Assessment of mindfulness by self-report: The Kentucky Inventory of Mindfulness Skills. Assessment. 2004;11(3):191-206.

43. Hatcher RL, Gillaspy JA. Development and validation of a revised short version of the Working Alliance Inventory. Psychotherapy Research. 2006;16(1):12-25.

44. MacKenzie KR. The clinical application of group measure. In: Dies RR, MacKenzie KR, editors. Advances in group psychotherapy: Integrating research and practice. New York: International Universities Press; 1983. p. 159-70.

45. Rahi S, Ghani M, Alnaser F, Ngah A. Investigating the role of unified theory of acceptance and use of technology (UTAUT) in internet banking adoption context. Management Science Letters. 2018;8(3):173-86.

46. Attkisson CC, Zwick R. The Client Satisfaction Questionnaire: Psychometric properties and correlations with service utilization and psychotherapy outcome. Evaluation and Program Planning. 1982;5(3):233-7.

47. Weiner BJ, Lewis CC, Stanick C, Powell BJ, Dorsey CN, Clary AS, et al. Psychometric assessment of three newly developed implementation outcome measures. Implementation Science. 2017;12:1-12.

48. Kien C, Griebler U, Schultes M-T, Thaler KJ, Stamm T. Psychometric testing of the German versions of three implementation outcome measures. Global Implementation Research and Applications. 2021;1(3):183-94.

49. Lazarus SA, Cheavens JS, Festa F, Rosenthal MZ. Interpersonal functioning in borderline personality disorder: A systematic review of behavioral and laboratory-based assessments. Clinical psychology review. 2014;34(3):193-205.

50. Mitchell AE, Dickens GL, Picchioni MM. Facial emotion processing in borderline personality disorder: a systematic review and meta-analysis. Neuropsychology review. 2014;24(2):166-84.

51. Lis S, Bohus M. Social interaction in borderline personality disorder. Current Psychiatry Reports. 2013;15:1-7.

52. Murphy RO, Ackermann KA, Handgraaf MJJ. Measuring Social Value Orientation. Judgment and Decision Making. 2011;6(8):771-8.

53. Lévay EE, Bajzát B, Unoka ZS. Expectation of selfishness from others in borderline personality disorder. Frontiers in Psychology. 2021;12:702227.

54. Lis S, Schaedler A, Liebke L, Hauschild S, Thome J, Schmahl C, et al. Borderline personality disorder features and sensitivity to injustice. Journal of personality disorders. 2018;32(2):192-206.

55. Baumert A, Beierlein C, Schmitt M, Kemper CJ, Kovaleva A, Liebig S, Rammstedt B. Measuring four perspectives of justice sensitivity with two items each. Journal of Personality Assessment. 2014;96(3):380-90.

56. Thome J, Liebke L, Bungert M, Schmahl C, Domes G, Bohus M, Lis S. Confidence in facial emotion recognition in borderline personality disorder. Personality Disorders: Theory, Research, and Treatment. 2016;7(2):159-68.

57. Kleindienst N, Hauschild S, Liebke L, Thome J, Bertsch K, Hensel S, Lis S. A negative bias in decoding positive social cues characterizes emotion processing in patients with symptom-remitted borderline personality disorder. Borderline Personality Disorder and Emotion Dysregulation. 2019;6:1-9.

58. Biermann M, Schulze A, Unterseher F, Hamm M, Atanasova K, Stahlberg D, Lis S. Trustworthiness judgments and Borderline Personality Disorder: an experimental study on the interplay of happiness and trustworthiness appraisals and the effects of wearing face masks during the Covid-19 pandemic in Germany. Borderline Personality Disorder and Emotion Dysregulation. 2022;9(1):27.

59. Schulze A, Rommelfanger B, Schendel E, Schott H, Lerchl A, Vonderlin R, Lis S. Attributional style in Borderline Personality Disorder is associated with self-esteem and loneliness. Borderline Personality Disorder and Emotion Dysregulation. in press.

60. Winter D, Herbert C, Koplin K, Schmahl C, Bohus M, Lis S. Negative evaluation bias for positive self-referential information in borderline personality disorder. PLoS One. 2015;10(1):e0117083.

61. Gutz L, Roepke S, Renneberg B. Cognitive and affective processing of social exclusion in borderline personality disorder and social anxiety disorder. Behaviour Research and Therapy. 2016;87:70-5.

62. Barton BB, Goerigk S, Wustenberg T, Dewald-Kaufmann J, Reinhard MA, Musil R, et al. Altered immediate behavioral response to partial social exclusion: A cross-diagnostic study in patients with borderline personality disorder and persistent depressive disorder. J Psychiatr Res. 2021;144:177-83.

63. Biermann M, Schulze A, Vonderlin R, Bohus M, Lyssenko L, Lis S. Shame, self-disgust, and envy: An experimental study on negative emotional response in borderline personality disorder during the confrontation with the own face. Frontiers in Psychiatry. 2023;14.

64. Lazarus SA, Cheavens JS. An examination of social network quality and composition in women with and without borderline personality disorder. Personal Disord. 2017;8(4):340-8.

65. Lazarus SA, Beeney JE, Howard KP, Strunk DR, Pilkonis PA, Cheavens JS. Characterization of relationship instability in women with borderline personality disorder: A social network analysis. Personal Disord. 2020;11(5):312-20.

66. Clifton A, Pilkonis PA, McCarty C. Social networks in borderline personality disorder. Journal of personality disorders. 2007;21(4):434-41.

67. Lazarus SA, Beeney JE, Howard KP, Strunk DR, Pilkonis P, Cheavens JS. Characterization of relationship instability in women with borderline personality disorder: A social network analysis. Personality Disorders: Theory, Research, and Treatment. 2020;11(5):312-20.

68. Ebner-Priemer UW, Trull TJ. Ambulatory assessment: An innovative and promising approach for clinical psychology. European Psychologist. 2009;14(2):109-19.

69. Trull TJ, Lane SP, Koval P, Ebner-Priemer UW. Affective dynamics in psychopathology. Emotion Review. 2015;7(4):355-61.

70. Santangelo PS, Reinhard I, Koudela-Hamila S, Bohus M, Holtmann J, Eid M, Ebner-Priemer UW. The temporal interplay of self-esteem instability and affective instability in borderline personality disorder patients’ everyday lives. Journal of Abnormal Psychology. 2017;126(8):1057-65.

71. McCool MW, Mochrie KD, Lothes JE, Guendner E, St. John J, Noel NE. Dialectical behavior therapy skills and urges to use alcohol and substances: An examination of diary cards. Substance Use & Misuse. 2023;58(11):1409-17.

72. Bitran AM, Hughes C, Bist J, Krall HR, Yin Q, Rizvi SL. The Effects of Dialectical Behavior Therapy on Joy: A Diary Card Study. (Preprint). 2025.

73. Bohus M, Deuschel T, Gimbel S, Görg N, Humm BG, Schüller M, Turan E, editors. Predicting premature termination of treatment in psychotherapy for borderline personality disorder. Proceedings of the Collaborative European Research Conference (CERC 2017), Karlsruhe, Germany; 2017.

74. Bohus M, Gimbel S, Goerg N, Humm BG, Schüller M, Steffens M, Vonderlin R, editors. Improving machine learning prediction performance for premature termination of psychotherapy. Artificial Intelligence: Methodology, Systems, and Applications: 18th International Conference, AIMSA 2018, Varna, Bulgaria, September 12–14, 2018, Proceedings 18; 2018: Springer.
